# Toll-Like Receptor 2-Independent Host Innate Immune Response against an Epidemic Strain of *Streptococcus suis* That Causes a Toxic Shock-Like Syndrome in Humans

**DOI:** 10.1371/journal.pone.0065031

**Published:** 2013-05-28

**Authors:** Claude Lachance, Mariela Segura, Pehuén Pereyra Gerber, Jianguo Xu, Marcelo Gottschalk

**Affiliations:** 1 Faculty of Veterinary Medicine, University of Montreal, Saint-Hyacinthe, Québec, Canada; 2 State Key Laboratory for Infectious Disease Prevention and Control, National Institute for Communicable Disease Control and Prevention, Chinese Center for Disease Control and Prevention, Beijing, China; University of São Paulo, Brazil

## Abstract

*Streptococcus suis* is an emerging zoonotic agent causing meningitis and septicemia. Outbreaks in humans in China with atypical cases of streptococcal toxic shock-like syndrome have been described to be caused by a clonal epidemic *S. suis* strain characterized as sequence type (ST) 7 by multilocus sequence typing, different from the classical ST1 usually isolated in Europe. Previous *in vitro* studies showed that Toll-like receptor (TLR) 2 plays a major role in *S. suis* ST1 interactions with host cells. In the present study, the *in vivo* role of TLR2 in systemic infections caused by *S. suis* ST1 or ST7 strains using TLR2 deficient (TLR2^−/−^) mice was evaluated. TLR2-mediated recognition significantly contributes to the acute disease caused by the highly virulent *S. suis* ST1 strain, since the TLR2^−/−^ mice remained unaffected when compared to wild type (WT) mice. The lack of mortality could not be associated with a lower bacterial burden; however, a significant decrease in the induction of pro-inflammatory mediators, as evaluated by microarray, real-time PCR and protein assays, was observed. On the other hand, TLR2^−/−^ mice infected with the epidemic ST7 strain presented no significant differences regarding survival and expression of pro-inflammatory mediators when compared to the WT mice. Together, these results show a TLR2-independent host innate immune response to *S. suis* that depends on the strain.

## Introduction

*Streptococcus suis* serotype 2 is a major swine pathogen and an important emerging zoonotic agent [1], [2]. In western countries, *S. suis* infections in humans have been usually restricted to workers in close contact with pigs or pork by-products. However, in South East and East Asia, this pathogen affects not only the population at risk, but also the general population, presenting a significant public health concern [3]. In fact, it has been shown that *S. suis* is the primary cause of adult meningitis in Vietnam, the secondary cause in Thailand and the tertiary cause in Hong Kong [4]–[6]. Two deadly human outbreaks of *S. suis* occurred in China within the last years, with the atypical characteristic of most patients presenting a streptococcal toxic shock-like syndrome (STSLS) that had rarely been reported beforehand [7]. Both outbreaks were caused by the same clonal epidemic *S. suis* strain, characterized as sequence type (ST) 7 by multilocus sequence typing (MLST), which is different from the classical highly virulent ST1 usually isolated in Europe [7].

Virulence factors as well as the pathogenesis of *S. suis* infection have partially been elucidated [8]. It is unknown how *S. suis*, despite its low quantities on mucosal surfaces, is able to traverse this first line of host defence to disseminate in the host and initiate disease. Survival of the organism once in the bloodstream is facilitated by the capsular polysaccharide, which efficiently hampers phagocytosis [8]. Furthermore, the hemolysin (suilysin) seems to protect bacteria against complement-mediated uptake and killing by neutrophils, macrophages and dendritic cells [9]. *S. suis* can thus be considered a truly extracellular systemic pathogen. In the event that *S. suis* fails to cause acute fatal septicemia, bacteria are able to reach the central nervous system via mechanisms that are only partially elucidated. It has been reported that *S. suis* interacts with brain microvascular endothelial cells and the choroid plexus epithelial cells to breach the blood-brain barrier [10], [11]. Either in the bloodstream or at the central nervous system, *S. suis* will elicit a rapid and exaggerated inflammatory immune response that has been associated with mortality and clinical signs of the disease [12]. Interestingly, differences in virulence exist between *S. suis* serotype 2 strains isolated in North America (different STs, intermediate virulence), Europe (ST1, highly virulent) and the ST7 strain responsible for the STSLS outbreak in China (epidemic strain) [13]. Although the exact virulence factors involved in such differences are still poorly understood, their virulence degree has been suggested to correlate with their respective capacity to induce exaggerated inflammation [14].

Toll-like receptors (TLRs) are critical sensors that activate the innate immune response [15], [16]. Once microbial ligands bind to these receptors, downstream signal transduction pathways are activated resulting in the upregulation and suppression of many genes, leading to the release of many cytokines and chemokines responsible for inflammation and sometimes damage to the host. On the other hand, pathogen recognition by these receptors may be essential to prevent failure of the innate immune system to detect traces of microorganisms before systemic invasion [17]. In the case of *S. suis*, *in vitro* studies performed with heat-killed whole cells or live bacteria of classical ST1 European strains showed that TLR2 is mainly implicated in cell activation after stimulation of different murine and human cells [18], [19]. More recently, *in vitro* studies carried out with the whole cells of the epidemic ST7 strain and human peripheral blood cells showed that not only TLR2 but also TLR6 and TLR9 play an important role on cell activation [20]. Since inflammation has been described as playing a fundamental role in the pathogenesis of the toxic shock-like syndrome caused by *S. suis* infection [3], it is then hypothesized that there are differences on the TLR2 *in vivo* activation pattern between strains of *S. suis* with different virulent potential. To investigate such hypothesis, mortality, bacterial load, and genes regulated in mice following experimental infections with either a highly virulent ST1 European strain or the Chinese epidemic ST7 strain were performed. Expression of important inflammatory mediators (proteins) was also measured. Results clearly showed a TLR2-dependent or -independent innate immune response depending on the strain responsible for the infection, suggesting different mechanisms of cell activation.

## Materials and Methods

### *S. suis* strains and growth conditions

*S. suis* serotype 2 strain P1/7 (highly virulent ST1), isolated from a case of meningitis in Europe [21] and SC84 (epidemic ST7), isolated from a case of STSLS in China [21] were used for experimental infections [22]. Both strains have already been sequenced [21]. Bacteria were grown as previously described in Todd-Hewitt broth (THB) [12]. Aliquots of bacterial suspension were plated using an Autoplate® 4000 (Spiral Biotech) onto sheep blood agar plates to accurately determine bacterial concentrations.

### Mice and experimental infections

Wild type (WT) 7-week-old female C57BL/6 mice or TLR2^−/−^ (B6.129-*Tlr2* ^tmlKir^/J) mice (Jackson Laboratory) were acclimatized to standard laboratory conditions with unlimited access to water and rodent chow. This study was carried out in strict accordance with the recommendations and approved by University of Montreal Animal Welfare Committee guidelines and policies (Permit Number: RECH-1570) in order to minimize suffering. On the day of the experiment, one ml of a bacterial suspension of 1×10^7^ CFU or the bacteria vehicle solution (sterile THB) was administrated by intraperitoneal injection. Optimal bacterial concentration was chosen based on previous studies with WT mice [23] and on preliminary experiments with TLR2^−/−^ mice (data not shown). Mice were euthanized at 6 h post-infection (p.i.) for the microarray, real-time RT-qPCR, and cytokines analysis. This time-point was chosen based on previous microarray studies of WT mice infected with the same strains [24]. For survival studies, clinical signs and mortality were monitored during 72 h. Dose of infection and p.i. times were selected based on preliminary results and previous work [23].

### Measurement of blood bacterial loads

Following infection, blood bacteremia was assessed in surviving mice by collecting a 5 μl blood sample from the tail up to day 6 p.i. Proper dilutions were plated and counted as described above.

### Collection, homogenization and extraction of spleen total RNA

At 6 h p.i., spleens were removed, cut in pieces and put in 1.5 mL of RNAlater solution (Qiagen) for stabilization of total RNA. Approximately 25 mg of spleen was then disrupted and homogenized in 600 μl of lysis buffer (Qiagen) using a rotor stator homogenizer (Tissue-tearor model 398, Biospec products). Total RNA from homogenized tissue was isolated and purified using an RNeasy mini kit with on-column DNase digestion (Qiagen) and kept at -80°C.

### Illumina microarray analysis

Microarray experiments were performed using the Illumina whole-genome expression beadchips technology platform (Illumina). Prior to the microarray experiment, the total RNA quality was assessed using the Agilent 2100 Bioanalyzer (Agilent Technologies). Microarray tests were performed according to manufacturer’s instructions. Total RNA was hybridized to the MouseRef-8 v.2 Illumina BeadChip. This BeadChip targets 25,697 RefSeq transcripts and covers over 19,100 unique genes. Sample positions on chips were randomly distributed.

Text files containing the signal and detection *P*-values per probe for each sample were imported into FlexArray software v.1.61 (McGill University and Genome Quebec Innovation Centre). Data were first raw-filtered and then further pre-processed by applying a lumi filter for normalizing data. An analysis of variance (ANOVA) was used to look for differentially expressed genes between infected and mock-infected groups, for TLR2^−/−^ and WT mice infected with either the ST1 or ST7 strain. In order to maintain manageable datasets, differentially expressed genes were defined by fold changes smaller or greater than 3-folds with an accompanying *P*-value ≤0.05.

### Validation of microarray data by qPCR

Eight genes were selected to validate microarray results by quantitative real-time RT-PCR (qPCR), which was executed to conform to the qPCR MIQE guidelines [25], [26]. Primers (Integrated DNA technologies) used for detection of genes were all verified to have reaction efficiencies between 90–110% (**Table 1**). Normalization of the data was done using the two most experimentally determined stable reference genes, Actin-β and β-2 microglobulin (β2m). Fold-change of gene expression was calculated using the normalized gene expression (ΔΔC_q_) calculation method of the CFX software manager v.2.1 (Bio-Rad). Samples from mock-infected WT mice were used as calibrator.

**Table 1:** Primer sequences used for real-time RT-qPCR.

Gene	Genebank ID	Amplicon Size	Forward Sequence	Reverse Sequence	Span Intron	PCR Efficiency
*Cxcl1 (Kc)*	NM_008176	101 bp	TCTCCGTTACTTGGGGACAC	CCACACTCAAGAATGGTCGC	Yes	103%
*Cxcl2 (Mip2a)*	NM_009140	102 bp	AACATCCAGAGCTTGAGTGTGA	TTCAGGGTCAAGGCAAACTT	Yes	100%
*Il6*	NM_031168	139 bp	ATGGATGCTACCAAACTGGAT	TGAAGGACTCTGGCTTTGTCT	Yes	103%
*Ifng*	NM_008337	90 bp	TGAGCTCATTGAATGCTTGG	ACAGCAAGGCGAAAAAGGAT	Yes	97%
*Ccl2 (Mcp1)*	NM_011333	108 bp	ATTGGGATCATCTTGCTGGT	CCTGCTGTTCACAGTTGCC	Yes	103%
*Ccl3 (Mip1a)*	NM_011337	91 bp	GTGGAATCTTCCGGCTGTAG	ACCATGACACTCTGCAACCA	Yes	95%
*Ccl4 (Mip1b)*	NM_013652	109 bp	GAAACAGCAGGAAGTGGGAG	CATGAAGCTCTGCGTGTCTG	Yes	95%
*Tnf*	NM_013693	103 bp	AGGGTCTGGGCCATAGAACT	CCACCACGCTCTTCTGTCTAC	Yes	99%
*Actinb*	NM_007393	170 bp	CCAACCGTGAAAAGATGACC	AGCATAGCCCTCGTAGATG	Yes	98%
*B2m*	NM_009735	110 bp	ATGGCTCGCTCGGTGACCCT	TTCTCCGGTGGGTGGCGTGA	Yes	99%

### Plasma cytokine and chemokine levels

Blood was collected from 6 h-euthanized mice and stabilized with EDTA. Time p.i. was chosen based on previous studies [12], [24]. Plasma supernatants were collected and kept at –80°C. Plasmatic concentrations of TNF, IL-6, IFN-γ, CCL2, CCL3, CCL4, CXCL1, and CXCL2 were determined using a custom-made 8-plex cytokine Milliplex panel (Millipore) according to manufacturer’s instructions. Acquisition was performed on a Luminex 100 platform (Luminex) and analysis performed using Beadview Multiplex Data Analysis Software v.1.0 (Upstate/Millipore).

### Statistical analysis

Data are presented as the mean ± standard error of the mean or geometric mean with 95% confidence interval where appropriate. Unpaired *t*-tests where appropriate were performed to find statistical differences between groups. The Kaplan–Meier method and log-rank Mantel-Cox tests were used to compare the survival rates of the studied groups. *P*<0.05 was considered statistically significant.

### Microarray data accession number

All microarray raw data are available and have been deposited in the Gene Omnibus Expression database under accession number GSE45861 and GSE45862.

## Results

### TLR2-dependent or –independent survival during *S. suis* infection

**Figure 1:**
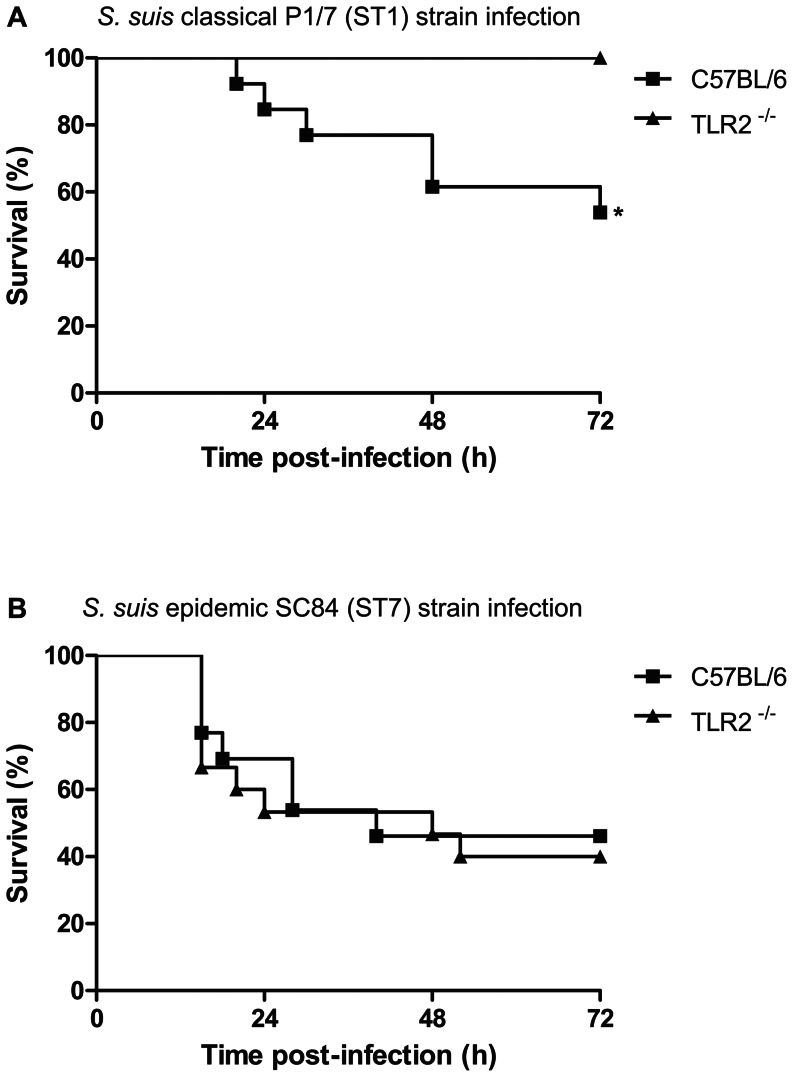
Absence of TLR2 increases survival following infection with *S.* *suis* highly virulent strain ST1 but not with the epidemic strain ST7. Survival of 7-week-old C57BL/6 mice (*n* = 13 mice per infection group) or TLR2^−/−^ mice (*n* = 14 mice per infection group) inoculated by intraperitoneal injection with 1×10^7^ CFU of either P1/7 strain (ST1; panel A) or SC84 strain (ST7; panel B) was monitored for 72 h. * *P*<0.01, indicates statistically significant differences between infected WT and TLR2^−/−^ mouse groups, as determined by Log-rank (Mantel-Cox) test.

**Figure 2:**
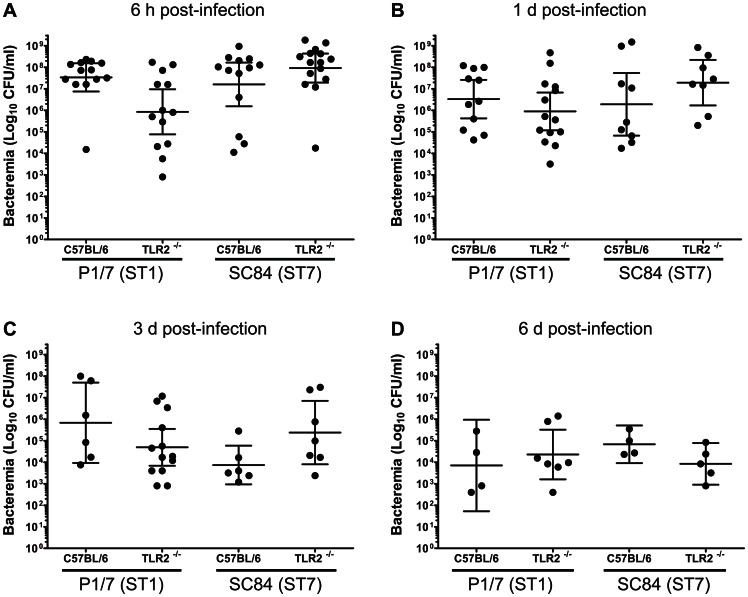
Blood bacteremia of *S.* *suis* highly virulent strain ST1 and epidemic strain ST7 is similar in C57BL/6 and TLR2^−/−^ mice. C57BL/6 mice (*n* = 13 mice per group) or TLR2^−/−^ mice (*n* = 14 mice per group) were inoculated by intraperitoneal injection with 1×10^7^ CFU of either *S. suis* strain P1/7 (ST1) or SC84 (ST7). At 6 h (panel A), day 1 (panel B), day 3 (panel C), and day 6 (panel D) post-infection, 5 μl of blood was harvested from the tail of infected mice and proper dilutions were plated on blood agar plate to assess blood bacteremia. Data of individuals are presented including geometric mean with 95% confidence interval. No significant differences (*P*>0.05) between infected TLR2^−/−^ mice and their WT counterpart were observed as determined by ANOVA.

As shown in **Figure 1A**, WT mice infected with the *S. suis* ST1 strain started dying at 20 h p.i. and 45% had succumbed by 72 h. On the other hand, TLR2^−/−^ mice infected with the same strain showed no mortality at the same time point (*P* = 0.004), even up to 96 h p.i. (data not shown). Survival of WT and TLR2^−/−^ mice after infection with the ST7 strain showed no significant differences (**Fig. 1B**). Indeed, WT and TLR2^−/−^ mice started dying at 16 h p.i. and 60% of mice had succumbed by 72 h (*P* = 0.745). These data confirmed the higher virulence of the ST7 strain compared to the ST1 strain, especially during the first hours of infection, as previously observed in C57BL/6 mice [24]. Results obtained suggest that mouse mortality after infection with the ST1 strain is, at least partially, related to the presence of TLR2. On the other hand, high mortality generated by the ST7 strain was shown to be independent of this receptor. Similar results were obtained when experiments were repeated (data not shown). WT and TLR2^−/−^ mice infected with either strain had similar levels of bacteremia (**Fig. 2**). Interestingly, mean levels of 10^4^ CFU/mL were uniformly observed 6 days p.i. for both strains in WT and TLR2^−/−^ mice, without the presence of clinical signs.

### Expression of several mouse genes after *S. suis* infection by the ST1 strain, but not by the ST7 strain, partially depends on TLR2

To investigate survival differences observed between WT and TLR2^−/−^ mice infected with the ST1 strain but not between mouse counterparts infected with the ST7 strain, a whole genome microarray study was undertaken. As shown in **Table S1**, several genes were upregulated in WT and TLR2^−/−^ mice at 6 h p.i. by both strains when compared to mock-infected mice. Several of these genes were related to proinflammatory cytokines, chemokines, and signaling pathways. Interestingly, this induction was generally lower in TLR2^−/−^ infected mice, particularly in mice infected with the ST1 strain. Many proinflammatory chemokines (such as CCL2, CCL3, CCL4, CCL7, CCL11, CXCL1, and CXCL2) were up-regulated to a lower level in TLR2^−/−^ mice infected with the ST1 strain than in WT counterparts. In contrast, no significant differences in the level of CCL2, CCL3, CCL4, CCL7, CCL11, CXCL1, and CXCL2 were observed between TLR2^−/−^ and WT mice infected with ST7 strain. Surprisingly, no significant differences in the up-regulation levels of IL-6 and TNF, two important cytokines involved in sepsis, were observed by microarray between TLR2^−/−^ and WT mice infected with either of the strains (**Table S1**).

**Figure 3:**
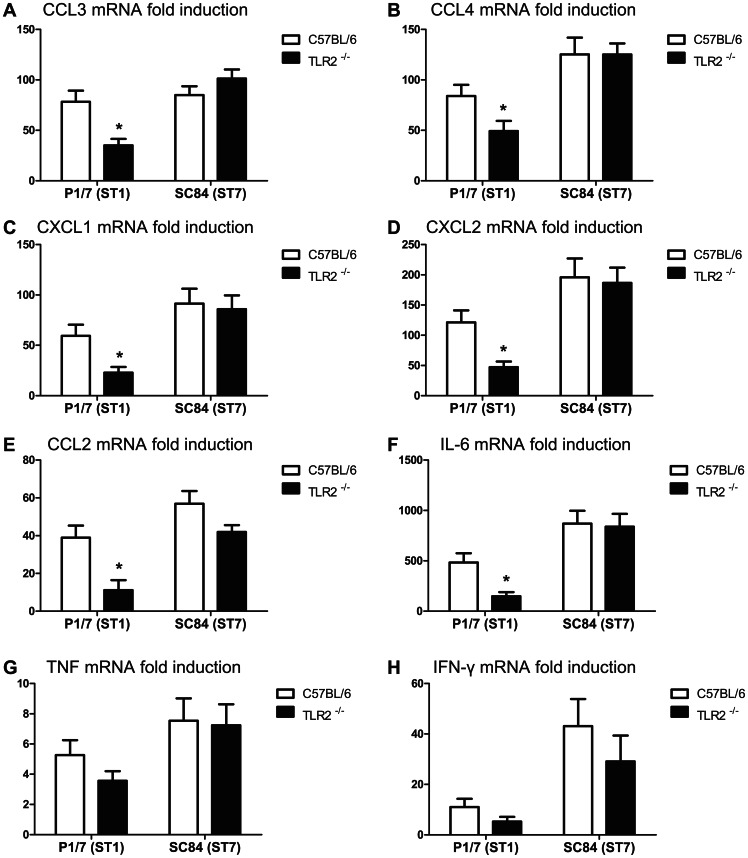
Proinflammatory cytokines and chemokines gene expression in TLR2^−/−^ or wild-type mice infected with ST1 or ST7 strain. Proinflammatory cytokine and chemokine gene expression in TLR2^−/−^ mice infected with *S. suis* highly virulent strain ST1 is lower than wild-type counterparts, whereas no major differences are observed between epidemic strain ST7-infected groups. Quantitative PCR analysis of cytokine and chemokine gene expression in C57BL/6 and TLR2^−/−^ mice infected with 1×10^7^ CFU of either *S. suis* strain P1/7 (ST1) or SC84 (ST7). Total RNA was isolated from spleen samples at 6 h post-infection. Tested cytokines and chemokines are indicated at the top of each panel. Data represent mean mRNA relative fold induction values ± SEM. * *P*<0.05, indicates TLR2^−/−^ group significantly different from corresponding C57BL/6 mice as determined by *t-test*.

Reduction in the expression levels of several proinflammatory genes in ST1-infected TLR2^−/−^ mice compared to WT counterparts, but not in those infected with the ST7 strain, was confirmed by qRT-PCR. A significant reduction in the levels of CCL3, CCL4, CXCL1, CXCL2, and CCL2 gene expression in TLR2^−/−^ mice infected with the highly virulent ST1 strain compared to WT infected mice was observed (**Fig. 3A**–**E**). Different from the results obtained by microarray, expression of IL-6 was also significantly reduced in ST1-infected TLR2^−/−^ mice compared to WT mice (**Fig. 3F**). In contrast, no significant differences were observed between ST7-infected groups of mice for these chemokines/cytokines (**Fig. 3A**–**F**). TNF gene up-regulation was not significantly different between groups of mice infected with either of the strains, confirming microarray results (**Fig. 3G**).

Production of high levels of IFN-γ has been associated to the STSLS induced by the ST7 strain [13], [24]. Expression of this cytokine was not significantly dependent on the presence of TLR2 for either strain of *S. suis* (**Fig. 3H**).

### ST1 and ST7 differences in TLR2 dependency are also observed at chemokine and cytokine protein levels

**Figure 4:**
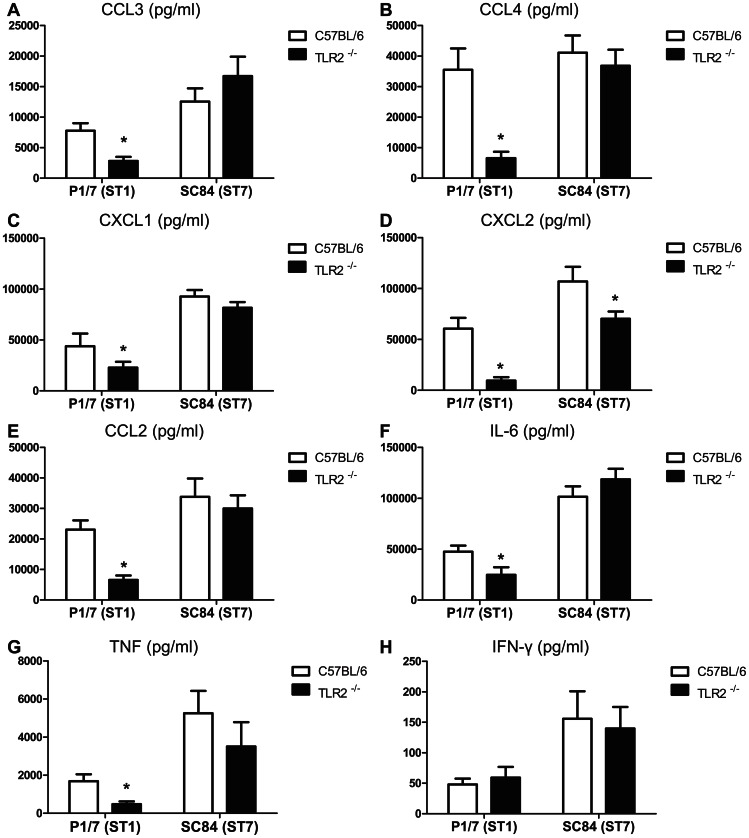
Production of proinflammatory cytokines and chemokines in TLR2^−/−^ or wild-type mice infected with ST1 or ST7 strain. Production of proinflammatory cytokines and chemokines in TLR2^−/−^ mice infected with *S. suis* highly virulent strain ST1 is lower than in wild type counterparts, whereas no differences are observed when animals are infected with epidemic strain ST7. Plasma levels of cytokines and chemokines in C57BL/6 and TLR2^−/−^ mice infected with 1×10^7^ CFU of *S. suis* strain P1/7 (ST1) or SC84 (ST7) for 6 h, as quantified by Luminex. Plasma samples were tested for the cytokines and chemokines indicated at the top of each panel. Data represent mean values ± SEM in pg/ml. * *P*<0.05 indicates statistically significant differences between infected TLR2^−/−^ group and its wild-type infected counterpart as determined by *t-*test.

Protein expression of proinflammatory chemokines and cytokines is tightly regulated at transcriptional and post-transcriptional levels. A Luminex assay was thus performed to confirm qPCR results. Similarly to qPCR and/or microarray results, protein levels of CCL2, CCL3, CCL4, CXCL1 and IL-6 were significantly lower only in TLR2^−/−^ mice infected with the ST1 strain than in WT counterparts (**Fig. 4A**–**C, E, F**). CXCL2 expression was also significantly different between these two groups of mice. However, a slight reduction of this chemokine expression was also noticeable in ST7-infected TLR2^−/−^ mice when compared to WT mice (**Fig. 4D**). Although not detected either by microarray or qPCR, TNF protein expression was clearly and significantly reduced in ST1-infected TLR2^−/−^ mice, whereas no significant changes were observed in mice infected with the ST7 strain (**Fig. 4G**). Protein expression of IFN-γ was also monitored and no significant differences were observed in infected TLR2^−/−^ mice when compared to WT mice, confirming gene expression results (**Fig. 4H**).

## Discussion

Inflammation plays an important role in the pathogenesis of *S. suis*-induced septicemia and meningitis [3]. Cases in humans and pigs with presence of shorter incubation time, more rapid disease progression, and a higher rate of mortality have been lately described and may be due, in part, to enhanced induction of inflammation. TLRs are critical sensors in detecting infections and initially activate the innate immune system. *S. suis* is a pathogen that, in acute bacterial infections, will induce an exacerbated inflammatory reaction that contributes to the disease [23]. Within the family of TLRs, TLR2 has been implicated as the major pattern recognition receptor for ligands derived from Gram-positive bacteria [27]–[29]. In the case of *S. suis*, several *in vitro* studies with ST1 strains have previously shown that TLR2 plays an important role during bacterial interactions with mouse, swine and human cells [18]–[20], [30]. In addition, *in vitro* data suggest that activation pattern of TLR of typical ST1 European strains and that responsible for the Chinese human outbreak of toxic shock-like syndrome may slightly differ [20]. However, the *in vivo* contribution of TLR2 to the pathology of *S. suis* acute systemic infections has so far never been studied.

Our data indicate that *in vivo* TLR2-mediated recognition significantly contributes to the severe outcome following infection with a highly virulent *S. suis* ST1 strain. The absence of mortality could not be associated with a lower bacterial burden, suggesting that TLR2 would not be directly implicated in bacterial killing. In fact, recent results showed that dendritic cells from myeloid differentiation factor 88 deficient mice (MyD88^−/−^) presented similar rates of phagocytosis and killing of *S. suis* serotype 2 than cells isolated from WT mice [31], suggesting that TLRs would probably not be implicated in *S. suis* clearance. On the other hand, microarray, qRT-PCR, and/or protein assays confirmed a significantly lower induction of proinflammatory cytokines and chemokines in TLR2^−/−^ mice that would correlate with survival, confirming previous *in vitro* results [18], [19]. Previous studies showed that the susceptibility of animals after experimental infection with *S. suis* serotype 2 clearly correlated with levels of inflammatory mediators in plasma rather than bacteremia [23]. Levels of proteins in plasma were similar to those obtained from homogenized spleen from infected mice (unpublished observations). *In vivo* results obtained with *S. suis* are similar to those reported by Mancuso *et al*. with Group B *Streptococcus* (GBS) who showed that, at high bacterial concentrations, TLR2^−/−^ mice presented similar levels of bacteremia but considerably lower levels of inflammatory mediators and less mortality than WT mice [17]. However, and differently from *S. suis*, TLR2 plays an important role in controlling GBS infection when mice are infected with low doses of bacteria [17]. Infections with lower *S. suis* concentrations (≤5×10^6^ CFU/mL) did not induce clinical disease in any of the mouse strains tested (results not shown). It is important to note that the pathogenesis of *S. suis* and GBS differ mainly in that the capsular polysaccharide of these pathogens present high or low anti-phagocytic properties, respectively [32].

Interestingly, inflammatory mediators were significantly reduced but not abolished in TLR2^−/−^ infected mice, suggesting the contribution of other receptors. *In vitro* studies with *S. suis* ST1 strains recently showed that TLRs other than TLR2 as well as non-TLR receptors, such as nucleotide-binding oligomerization domain-like receptor (NLR) families, TLR3, TLR6 and TLR9 may also be involved in cell activation [31]. In fact, mice infected with very high doses of both *S. suis* ST1 and ST7 (≥ 1×10^8^ CFU/mL) died within 72 h of infection indicating that although TLR2 seems to play an important role, *in vivo* activation of host cells by *S. suis* would require a multimodal recognition system (results not shown).

Serious outbreaks of *S. suis* STSLS in humans caused by an atypical strain have been reported during the last few years in China [33]. The ST7 strain was already shown to possess both higher inflammatory and virulence capacities than those of European and North American strains [22]. So far, the production of superantigens could not be proved for the epidemic strain [13]. However, a recent report showed some differences in receptor recognition *in vitro* between these two strain types [20]. Results observed in the present study indicate that TLR2 does not seem to be mainly implicated in the severe symptoms and lethality associated with the ST7 strain. Bacterial loads and induction of most proinflammatory cytokines and chemokines were independent of TLR2. We have recently reported that the higher mortality observed with the ST7 strain compared to ST1 strain was mainly due to a massive secretion of IFN-γ by Natural killer cells [24]. In the present study, levels of mRNA and protein of IFN-γ were significantly higher in ST7 compared to ST1-infected mice (*P* <0.05), confirming our previous observations. However, levels of this mediator were induced at similar levels by WT and TLR2^−/−^ ST7-infected mice, which may explain, at least in part, similar levels of mortality in both mouse groups. It is quite possible that this particular strain of *S. suis* has a better capacity to stimulate other host receptors besides TLR2 during a systemic infection. As mentioned above, other TLR receptors (such as TLR3, TLR6 and TLR9) as well as other non-TLR receptors, such as NLR families, may also be involved in cell activation of this particular strain. So far, virulence factors that would be present in this strain (but absent in ST1 strains) that may be responsible for such differences have not been clearly identified [8]. The implication of lipoteichoic acid (LTA) in the activation of TLRs is still controversial [29], [34]. For GBS for example, it has been clearly demonstrated that secreted lipoproteins but not LTA are essential for TLR2 activation [35]. Cell-wall associated lipoproteins of *S. suis* have been suggested as being implicated on TLR2/6 but not TLR1/2 cell activation and this effect varies depending on the strain and serotype [30]. Further studies using isogenic mutants clearly demonstrated that lipoproteins are potent and dominant innate immunity activating molecules of *S. suis* [36]. These lipoproteins can be released after penicillin treatment or directly released in the supernatant [36]. However, receptors involved in the activation of these released bacterial components have not been elucidated. A recent study showed that differences on *in vitro* TLR activation between an European ST1 and the ST7 epidemic strain were observed also with washed heat killed suspensions, indicating a lower implication of secreted components [20]. Thus, we hypothesize that different bacterial components, especially differences in lipoproteins (secreted or not) present in typical *S. suis* strains and those from the epidemic *S. suis* ST7 strain may vary and play a distinct role on cell activation and in the pathogenesis of the systemic inflammatory disease caused by this pathogen. Results obtained in the present study with both ST1 and ST7 strains reinforce the concept of multiple Gram-positive cell receptors. Further investigations using different genetic mouse models defective in single TLRs, MyD88 or with double TLR deletions will help clarify the role of other receptors in the innate recognition of typical ST1 *S. suis* strains but mainly, the epidemic *S. suis* ST7 strain.

Results obtained in this study can be applied to the acute systemic infection caused by *S. suis*. However, *S. suis* is also able to induce meningitis in a mouse model of infection at later incubation times (between 5 and 14 days post-infection) [12]. Some cases of meningitis have also been induced in humans by the ST7 strain during the outbreak in China [22]. In fact, the actual *in vivo* role of TLR2 in meningitis caused by any strain of *S. suis* is unknown, and it would be difficult to predict. For example, it has been reported that TLR2 does not play a major role in *Streptococcus pneumoniae* killing and disease after either systemic disease or pneumonia [37], [38]. However, other studies showed that TLR2^−/−^ mice are significantly more affected and have increased bacterial loads than WT mice in experimental meningitis [39], [40]. Although TLR2 has been suggested to be implicated in *S. suis* meningitis [41], further *in vivo* studies with TLR2^−/−^ mice are warranted.

In summary, results obtained in this study reveal that infection of mice by highly pathogenic strains of *S. suis* may follow TLR2-dependent or independent pathways depending on the strain. The atypical epidemic ST7 strain, responsible for STSLS human cases, would not only induce a massive and distinctive IFN-γ response but also activate cells using currently unknown receptors which are different from those activated by highly virulent ST1 strains.

## Supporting Information

Table S1 **Genes upregulated greater than three-fold in wild type C57BL/6 (B6) or TLR2** ^−/−^ **mice infected with either** ***S. suis*** **P1/7 (ST1) strain or epidemic SC84 (ST7) strain for 6 h.**
(DOCX)
