# Supplementary material for: Toll-Like Receptor 2-Independent Host Innate Immune Response against an Epidemic Strain of Streptococcus suis That Causes a Toxic Shock-Like Syndrome in Humans
Source: PLoS One. 2013 May 28;8(5):e65031. doi: 10.1371/journal.pone.0065031 (PMC3665724; doi:10.1371/journal.pone.0065031)
Supplement: Table S1 — Genes upregulated greater than three-fold in wild type C57BL/6 (B6) or TLR2 −/− mice infected with either S. suis P1/7 (ST1) strain or epidemic SC84 (ST7) strain for 6 h. (DOCX) [file pone.0065031.s001.docx]

Supplementary Table S1

Genes upregulated greater than three-fold in wild type C57BL/6 (B6) or TLR2^-/-^ mice infected with either *S. suis* P1/7 (ST1) strain or epidemic SC84 (ST7) strain for 6 h.

| Genebank ID | | Gene | Gene description | P1/7  (ST1) (B6) | P1/7  (ST1)  (TLR2^-/-^) | SC84  (ST7) (B6) | SC84  (ST7)  (TLR2^-/-^) |
| --- | --- | --- | --- | --- | --- | --- | --- |
|  | **Cytokines, chemokines, and related receptors** | | | | | | |
| NM_011333.3 | | *Ccl2* | Chemokine (C-C motif) ligand 2, MCP-1 | **3.7** | 2.2 | **7.2** | **5.9** |
| NM_011337.2 | | *Ccl3* | Chemokine (C-C motif) ligand 3, MIP-1α | **28.5** | **11.9** | **46.7** | **35.1** |
| NM_013652.2 | | *Ccl4* | Chemokine (C-C motif) ligand 4, MIP-1β | **38.0** | **12.6** | **53.7** | **35.6** |
| NM_013654.2 | | *Ccl7* | Chemokine (C-C motif) ligand 7, MCP-3 | **3.8** | 2.0 | **11.7** | **15.5** |
| NM_011330.2 | | *Ccl11* | Chemokine (C-C motif) ligand 11, Eotaxin | **8.5** | **3.6** | **12.4** | **11.6** |
| NM_017466.4 | | *Ccrl2* | Chemokine (C-C motif) receptor-like 2, Cmkbr1l2 | **5.7** | **3.8** | **10.7** | **9.6** |
| NM_008176.1 | | *Cxcl1* | Chemokine (C-X-C motif) ligand 1, KC | **19.8** | **11.3** | **35.4** | **29.2** |
| NM_009140.2 | | *Cxcl2* | Chemokine (C-X-C motif) ligand 2, MIP-2α | **6.6** | **3.5** | **16.4** | **13.6** |
| NM_008599.3 | | *Cxcl9* | Chemokine (C-X-C motif) ligand 9, MIG | 2.9 | 2.1 | **5.6** | **4.4** |
| NM_011819.1 | | *Gdf15* | Growth differentiation factor 15 | 2.9 | 1.7 | **3.7** | **4.3** |
| NM_008337.3 | | *Ifng* | Interferon-γ | 2.7 | 1.7 | **10.9** | **6.9** |
| NM_010554 | | *Il1a* | Interleukin 1α | 3.0 | 2.0 | **4.6** | **4.9** |
| NM_008361 | | *Il1b* | Interleukin 1β | 2.5 | 1.7 | **4.3** | 2.4 |
| NM_031167.3 | | *Il1rn* | Interleukin 1 receptor antagonist, transcript 1 | **7.1** | **3.9** | **9.1** | **9.7** |
| NM_031168.1 | | *Il6* | Interleukin 6 | 2.2 | 1.8 | **6.0** | **4.5** |
| NM_010548.1 | | *Il10* | Interleukin 10 | 2.6 | 2.5 | **3.9** | **3.5** |
| NM_145636.1 | | *Il27* | Interleukin 27 | 1.2 | 2.0 | **3.9** | **3.2** |
| NM_133775.1 | | *Il33* | Interleukin 33 | 2.1 | 1.9 | **3.2** | **3.0** |
| NM_008694.1 | | *Ngp* | Neutrophilic granule protein | 2.3 | **3.2** | **3.2** | **3.1** |
| NM_013693.1 | | *Tnf* | Tumor necrosis factor | **4.8** | **3.7** | **8.0** | **7.7** |
| NM_009396.1 | | *Tnfaip2* | Tumor necrosis factor, α-induced protein 2 | 2.7 | 2.2 | **3.3** | **3.4** |
| NM_009397.2 | | *Tnfaip3* | Tumor necrosis factor, α-induced protein 3 | **4.3** | **3.7** | **6.2** | **4.6** |
| NM_011659.2 | | *Tnfrsf4* | Tumor necrosis factor receptor superfamily, member 4 | 2.7 | 2.5 | **3.3** | **3.4** |
|  | **Cytokine signalling** | | | | | | |
| NM_031185.2 | | *Akap12* | A kinase (PRKA) anchor protein (gravin) 12 | **9.6** | **5.7** | **10.5** | **10.4** |
| NM_016847.2 | | *Avpr1a* | Arginine vasopressin receptor 1A | 2.9 | **3.2** | **3.7** | **3.8** |
| NM_009895.3 | | *Cish* | Cytokine inducible SH2-containing protein | 2.7 | 2.1 | **4.7** | **3.2** |
| NM_013642.2 | | *Dusp1* | Dual specificity phosphatase 1 | **5.8** | **4.0** | **6.8** | **5.5** |
| NM_130447.2 | | *Dusp16* | Dual specificity phosphatase 2 | 1.0 | 2.3 | **3.1** | 2.7 |
| NM_010104.2 | | *Edn1* | Endothelin 1 | **4.1** | 2.4 | **5.1** | **5.3** |
| NM_133753.1 | | *Errfi1* | ERBB receptor feedback inhibitor 1 | **7.3** | **5.0** | **8.2** | **6.0** |
| NM_173398.2 | | *Gpr171* | G protein-coupled receptor 171 | **3.5** | **3.6** | **4.7** | **4.5** |
| NM_133662.2 | | *Ier3* | Immediate early response 3 | 2.9 | 2.2 | **4.5** | **4.2** |
| NM_008390.1 | | *Irf1* | Interferon regulatory factor 1 | 2.2 | 1.7 | **4.1** | **3.0** |
| NM_016850.2 | | *Irf7* | Iinterferon regulatory factor 7 | 2.9 | 1.8 | **4.3** | 2.4 |
| NM_181593.2 | | *Itpkc* | Inositol 1,4,5-trisphosphate 3-kinase C | 2.3 | 2.1 | **3.4** | **3.1** |
| NM_007746.2 | | *Map3k8* | Mitogen-activated protein kinase kinase kinase 8 | 2.9 | 2.6 | **3.2** | 2.9 |
| NM_172603.2 | | *Phf11* | PHD finger protein 11 | 2.6 | 1.6 | **3.4** | 2.1 |
| NM_015811.1 | | *Rgs1* | Regulator of G-protein signaling 1 | **6.8** | **3.8** | **8.8** | **6.3** |
| NM_011267.2 | | *Rgs16* | Regulator of G-protein signaling 16 | 2.2 | 1.5 | **3.4** | 2.8 |
| NM_001033335.2 | | *Serpina3f* | Serine peptidase inhibitor, clade A, member 3F | **6.1** | **4.4** | **8.3** | **5.5** |
| NM_007707.2 | | *Socs3* | Suppressor of cytokine signaling 3 | **4.9** | **4.3** | **6.0** | **5.1** |
| NM_146162.1 | | *Tmem119* | Transmembrane protein 119 | 2.5 | 1.7 | **3.0** | 2.3 |
| NM_023137.2 | | *Ubd* | Ubiquitin D | **3.0** | 2.0 | **5.2** | **3.9** |
|  | **Host defense** | | | | | | |
| NM_009841.3 | | *Cd14* | CD14 | **5.6** | **5.7** | **8.3** | **7.8** |
| NM_013459.1 | | *Cfd* | Complement factor D (adipsin) | 1.0 | 0.4 | **3.3** | 2.4 |
| NM_010819.3 | | *Clec4d* | C-type lectin domain family 4, member d | 2.4 | 1.6 | **3.3** | **3.5** |
| NM_019948.2 | | *Clec4e* | C-type lectin domain family 4, member e | 2.2 | 2.1 | **3.4** | **3.3** |
| NM_030150.2 | | *Dhx58* | DEXH (Asp-Glu-X-His) box polypeptide 58 | 2.9 | 1.7 | **3.6** | 2.2 |
| NM_144559.1 | | *Fcgr4* | Fc receptor, IgG, low affinity IV | 2.8 | 2.1 | **3.3** | 2.6 |
| NM_008332.2 | | *Ifit2* | Interferon-induced protein with tetratricopeptide repeats 2 | **4.2** | **2.2** | **7.7** | **4.7** |
| NM_010501.2 | | *Ifit3* | Interferon-induced protein with tetratricopeptide repeats 3 | **5.4** | 2.1 | **6.4** | **3.4** |
| NM_008330.1 | | *Ifi47* | Interferon-γ inducible protein 47 | 2.7 | 1.7 | **3.1** | 2.7 |
| NM_008326.1 | | *Irgm1* | Immunity-related GTPase family M member 1 | **3.6** | 1.0 | **5.3** | **3.6** |
| NM_020583.4 | | *Isg20* | Interferon-stimulated protein 20 | 2.0 | 1.3 | **3.3** | 1.8 |
| NM_008491.1 | | *Lcn2* | Lipocalin 2 | **3.9** | **3.8** | **4.7** | **4.1** |
| NM_010846.1 | | *Mx1* | Myxovirus (influenza virus) resistance 1 | 2.0 | 1.6 | **3.0** | 2.3 |
| NM_013606 | | *Mx2* | Myxovirus (influenza virus) resistance 2 | **4.3** | 2.2 | **7.4** | **3.6** |
| NM_011852.2 | | *Oas1g* | 2'-5' oligoadenylate synthetase 1G | 2.4 | 1.3 | **3.0** | 2.3 |
| NM_145227.1 | | *Oas2* | 2'-5' oligoadenylate synthetase 2 | 2.6 | 1.8 | **3.7** | 2.4 |
| NM_145209.2 | | *Oasl1* | 2'-5' oligoadenylate synthetase-like 1 | **5.2** | 2.3 | **7.0** | **4.7** |
| NM_011854.1 | | *Oasl2* | 2'-5' oligoadenylate synthetase-like 2 | 2.1 | 1.3 | 2.7 | 1.8 |
| NM_008987.3 | | *Ptx3* | Pentraxin related gene | 1.4 | 1.5 | **4.7** | **3.8** |
| NM_021384.3 | | *Rsad2* | Radical S-adenosyl methionine domain containing 2 | **5.8** | 1.1 | **8.3** | 2.6 |
| NM_018851.2 | | *Samhd1* | SAM domain and HD domain, 1 | 2.1 | 1.2 | **3.0** | 1.4 |
|  | **Apoptosis, cell cycle regulation, and oncogenesis** | | | | | | |
| NM_153287.3 | | *Axud1* | AXIN1 up-regulated 1 | 2.6 | 1.9 | **3.5** | **3.6** |
| NM_207680.2 | | *Bcl2l11* | BCL2-like 11 (apoptosis facilitator), transcript variant 1 | 2.9 | 2.8 | **3.2** | 2.9 |
| NM_007829.3 | | *Daxx* | Fas death domain-associated protein | **3.3** | 2.0 | **4.7** | **3.2** |
| NM_010128.4 | | *Emp1* | Epithelial membrane protein 1 | 4.3 | 2.3 | **5.6** | **4.9** |
| NM_011046.2 | | *Furin* | Furin (paired basic amino acid cleaving enzyme) | 2.2 | 1.9 | **3.6** | 1.0 |
| NM_008655.1 | | *Gadd45b* | Growth arrest and DNA-damage-inducible 45β | **3.2** | 2.4 | **3.4** | **3.7** |
| NM_030701.1 | | *Gpr109a* | G protein-coupled receptor 109A | 2.8 | 2.0 | **4.2** | **4.3** |
| NM_010807.3 | | *Marcksl1* | MARCKS-like 1 | 2.7 | 1.7 | **3.5** | **3.2** |
| NM_008654.1 | | *Myd116* | Myeloid differentiation primary response gene 116 | 2.7 | 1.3 | **4.0** | 1.9 |
| NM_009344.1 | | *Phlda1* | Pleckstrin homology-like domain, family A, member 1 | 1.9 | 1.6 | **3.4** | 2.3 |
| NM_152804.1 | | *Plk2* | Polo-like kinase 2 | 2.6 | 2.0 | **3.3** | **3.3** |
|  | **Transcriptional and translational regulation** | | | | | | |
| NM_028967.1 | | *Batf2* | Basic leucine zipper transcription factor, ATF-like 2 | 1.9 | 1.7 | **3.5** | 2.7 |
| NM_009883.3 | | *Cebpb* | CCAAT/enhancer binding protein (C/EBP), β | 2.7 | **3.0** | **4.0** | **3.3** |
| NM_013498.1 | | *Crem* | cAMP responsive element modulator | 2.2 | 1.9 | **3.8** | **3.1** |
| NM_199015.1 | | *D14ertd668e* | DNA segment, Chr 14, ERATO Doi 668, expressed | **3.1** | 1.6 | **3.4** | 2.4 |
| NM_011163.3 | | *Eif2ak2* | Eukaryotic translation initiation factor 2-α kinase 2 | **3.1** | 1.8 | **3.3** | **3.0** |
| NM_010234.2 | | *Fos* | FBJ osteosarcoma oncogene | 2.6 | 2.6 | **4.6** | **4.1** |
| NM_008416.1 | | *Junb* | Jun-B oncogene | 2.7 | 2.7 | **3.9** | **3.2** |
| NM_010907.1 | | *Nfkbia* | Nuclear factor of kappa light polypeptide gene enhancer in B-cells inhibitor, α | **3.5** | 2.8 | **4.0** | **3.5** |
| NM_030612.2 | | *Nfkbiz* | nuclear factor of kappa light polypeptide gene enhancer in B-cells inhibitor, β | **4.2** | **4.2** | **5.2** | **5.6** |
| NM_001039530.1 | | *Parp14* | Poly (ADP-ribose) polymerase family, member 14 | **3.1** | 2.4 | **3.9** | **3.4** |
|  | **Fatty acid metabolism** | | | | | | |
| NM_009890.1 | | *Ch25h* | Cholesterol 25-hydroxylase | 2.4 | 1.7 | **3.5** | 2.9 |
| NM_011636.1 | | *Plscr1* | Phospholipid scramblase 1 | 2.9 | 2.2 | **3.0** | 2.6 |
| NM_011315.3 | | *Saa3* | Serum amyloid A 3 | **3.7** | **6.1** | **6.6** | **6.9** |
| NM_011451.2 | | *Sphk1* | Sphingosine kinase 1 (Sphk1), transcript variant 1 | **7.5** | **3.7** | **8.3** | **5.9** |
|  | **Cell adhesion and migration** | | | | | | |
| NM_172845.1 | | *Adamts4* | ADAM metallopeptidase with thrombospondin type 1 motif, 4 | **5.4** | **4.0** | **10.1** | **6.6** |
| NM_008607.1 | | *Mmp13* | Matrix metallopeptidase 13 | **4.1** | **3.1** | **7.0** | **3.1** |
| NM_008608.2 | | *Mmp14* | Matrix metallopeptidase 14 | **3.0** | 2.6 | **3.1** | 2.9 |
| NM_010809.1 | | *Mmp3* | Matrix metallopeptidase 3 | **3.8** | 2.3 | **5.5** | **3.9** |
| NM_008630.2 | | *Mt2* | Metallothionein 2 | 2.8 | 2.5 | **3.2** | 2.4 |
| NM_011113.3 | | *Plaur* | Plasminogen activator, urokinase receptor | 2.4 | 1.9 | **3.1** | **3.1** |
| NM_011593.2 | | *Timp1* | Tissue inhibitor of metalloproteinase 1, transcript variant 2 | **6.0** | **3.6** | **8.3** | **6.7** |
|  | **Surface receptor molecule, antigen presentation and co-stimulation** | | | | | | |
| NM_021893.2 | | *Cd274* | CD274 | **5.1** | **3.6** | **5.9** | **6.0** |
| NM_001033122.3 | | *Cd69* | CD69 | **4.8** | **3.3** | **6.8** | **4.4** |
|  | **Angiogenesis** | | | | | | |
| NM_007426.3 | | *Angpt2* | Angiopoietin 2 | **3.3** | 2.3 | **3.0** | **4.2** |
| NM_010415.1 | | *Hbegf* | Heparin-binding EGF-like growth factor | 2.5 | 1.7 | **3.9** | 2.9 |
|  | **Cytoskeleton/actin rearrangement** | | | | | | |
| NM_007930.3 | | *Enc1* | Ectodermal-neural cortex 1 | **3.1** | 2.5 | **4.1** | **3.5** |
| NM_007984.2 | | *Fscn1* | Fascin homolog 1, actin bundling protein | 2.8 | 2.3 | **4.4** | 2.6 |
| NM_026473.2 | | *Tubb6* | Tubulin, β6 | 2.6 | 2.5 | **3.1** | **4.1** |
|  | **Biological and metabolic process** | | | | | | |
| NM_024495.4 | | *Car13* | Carbonic anhydrase 13 | 2.9 | 2.1 | **3.3** | **3.2** |
| NM_007669.3 | | *Cdkn1a* | Cyclin-dependent kinase inhibitor 1A | **6.7** | **4.5** | **10.2** | **6.5** |
| NM_007695.2 | | *Chi3l1* | Chitinase 3-like 3 | 2.5 | 1.9 | **3.2** | **3.0** |
| NM_010260.1 | | *Gbp2* | Guanylate binding protein 2 | **3.5** | 2.4 | **5.3** | **3.5** |
| NM_018734.2 | | *Gbp3* | Guanylate binding protein 3 | 2.3 | 1.7 | **3.6** | 2.0 |
| NM_153564.2 | | *Gbp5* | Guanylate binding protein 5 | 2.4 | 1.8 | **4.2** | **3.1** |
| NM_008102.3 | | *Gch1* | GTP cyclohydrolase 1 | 1.2 | 1.2 | **3.2** | 2.3 |
| NM_008230.4 | | *Hdc* | Histidine decarboxylase | 2.7 | 2.6 | **3.4** | **4.2** |
| NM_018738.3 | | *Igtp* | Interferon gamma induced GTPase | **3.2** | 1.9 | **4.2** | **3.0** |
| NM_021342.1 | | *Kcne4* | Potassium voltage-gated channel, Isk-related subfamily, gene 4 | 2.0 | 1.8 | **3.1** | 2.8 |
| NM_153101.1 | | *Mrgpra2* | MAS-related GPR, member A2 | 2.9 | 2.2 | **3.2** | **4.0** |
| NM_026835.2 | | *Ms4a6d* | membrane-spanning 4-domains, subfamily A, member 6D | 2.8 | 2.5 | **3.3** | 2.7 |
| NM_013614.1 | | *Odc1* | ornithine decarboxylase, structural 1 | **4.3** | 2.1 | **4.7** | 2.9 |
| NM_019511.3 | | *Ramp3* | Receptor (calcitonin) activity modifying protein 3 | 2.1 | 1.8 | **4.2** | **4.6** |
| NM_023044.1 | | *Slc15a3* | Solute carrier family 15, member 3 | 2.5 | 2.5 | **3.3** | **3.5** |
| NM_011990.2 | | *Slc7a11* | Solute carrier family 7, member 11 | **3.4** | 2.4 | **4.2** | **4.3** |
| NM_020557.3 | | *Tyki* | Cytidine monophosphate (UMP-CMP) kinase 2 | 2.9 | 1.5 | **4.0** | **3.3** |
| NM_009477.1 | | *Upp1* | Uridine phosphorylase 1 | **3.9** | **3.2** | **4.0** | **5.0** |
| NM_011909.1 | | *Usp18* | Ubiquitin specific peptidase 18 | **6.0** | 2.1 | **8.1** | **4.6** |

Values represent mean fold increase compared to their counterpart mock-infected TLR2^-/-^ or C57BL/6 mice, as determined by Illumina microarray analysis.
